# Southern Dental Association

**Published:** 1869-09

**Authors:** Jno. G. Angell

**Affiliations:** Recording Secretary.


### ARTICLE III.

### *Southern Dental Association.*

Minutes of the Meeting of the Delegation of Southern Dentists held at Atlanta, Ga., July 28th, 29th and 30th, 1869.

By Jno. G. Angell, D.D.S., Recording Secretary.

A number of Southern Dentists met in Atlanta, Ga., for the purpose of organizing a Southern Dental Association; and the City Hall being tendered to them to hold their meeting they met in that building on the morning of July 29th, 1869.

The meeting was called to order by Dr. W. T. Arrington, of Memphis, Tenn.

On motion of Dr. C. A. Jordan, of Huntsville, Ala., Dr. Jas. S. Knapp, of New Orleans, was elected temporary President of the meeting.

On motion of Dr. W. T. Arrington, Dr. W. H. Morgan, of Nashville, Tenn., was elected Temporary Vice President, Drs. F. J. S. Gorgas, of Baltimore, Md. Temporary Secretary, and John G. Angell, of New Orleans, La. Assistant Secretary.

.Dr. Morgan moved then that if it was the sense of the convention here assembled, we proceed to organize a Dental Association, which was carried by a unanimous vote.

On motion of Dr. W. T. Arrington a committee of three was appointed to draft a Constitution and By-Laws for the Association, consisting of Drs. Morgan, W. T. Arrington and W. G. Redman, of Louisville Ky.

On motion of Dr. Morgan the following gentlemen were added to the committee: Drs. W. S. Chandler of New Orleans, La.; F. J. S. Gorgas of Baltimore and Thos. J. Jones, of Sparta, Ga. The committee then retired.

The following communication was read before the Convention : "The members of the Convention are respectfully invited to visit the Atlanta Medical College during their stay in the city. The exercises continue from 8 to 12 o'clock and from 2 to 4 in the afternoon. Signed, J. G. Westmoreland."

Various members exchanged views upon the object of the meeting and what should be the status of a delegate to become a member. The time was thus occupied until the Committee on Constitution and By-Laws appeared with their report,

The Committee submitted a constitution, which, on being read was received as a whole to be subsequently adopted article by article.

In the second reading Article 1st and 2nd were adopted as read. Articles 3rd and 4th met with so many objections, that each member was allotted five minutes to give his views, when it was found there was so much dissatisfaction about these two articles, they were, on motion of Dr. Morgan, recommitted to the committee with instructions to report at the afternoon session.

On motion of Dr. Morgan, the convention continued to consider the remaining articles of the constitution which were adopted as read. The convention then adjourned until 3 P.M.

AFTERNOON SESSION.

The meeting was called to order by Dr. Jas. S. Knapp, the President, at 3 P. M. as per adjournment.

The Committee on Constitution and By-Laws not being ready to report, on motion of Dr. Angell the Code of Ethics was taken under consideration during their absence, and on being read was adopted as a whole. The Committee on Constitution and By-Laws appeared, and not being able to agree as to proper qualifications for membership reported in favor of none taking part in the proceedings of the meetings except those who were balloted for and received three-fourth favorable votes. On motion of Dr. B. F. Arrington the report of the Committee was received and laid on the table. The following resolution was then offerred and accepted as a substitute to Articles 3. and 4.

Resolved that membership in this Association shall consist of such only as shall have received a degree in Dentistry or Medicine, and are engaged in the practice of Dentistry, or those who shall have been engaged in the practice ten years prior to the present time and received a three-fourth favorable vote of the members present to elect.

On motion of Dr. W. T. Arrington, the Constitution as a whole was adopted. The meeting then adjourned until eight P. M.

NIGHT SESSION.

As per adjournment the convention met at 8 P. M. and was called to order by the President, Dr. J. S. Knapp.

On motion of Dr. J. G. Angell a recess of one-half hour was granted to give the delegates an opportunity of signing the constitution and pay their initiation fee. The following are the names of the delegates who signed the Constitution and paid their fee :

Jas. S. Knapp, D. D. S., M. D., New Orleans, La.; W. H. Morgan, M. D., D. D. S., Nashville, Tenn.; W. T. Arrington, D. D. S., Memphis, Tenn.; J. R. Walker, D. D. S., N. Orleans, La.; H. Marshall, Atlanta, Geo.; W. S. Chandler, D.D.S., New Orleans, La.; Samuel Rambo, M. D.,D.D.S., Montgomery, Ala.; Henry A. Lowrance, Athens, Ga.; Arthur W. Ford, Atlanta, Geo.; R. A. McDonald, Griffin, Geo.; J. P. H. Brown, Augusta, Geo.; Thos. J. Jones, D. D. S., Sparta, Geo.; H. I. Henry, Covington, Geo.; J. A. Tigner, Fort Valley, Geo.; Edwin W. L'Engle, D. D. S., Savanah, Ga.; W. G. Redman, D.D.S., Louisville, Ky.; S. G. Holland, Augusta, Geo.; Jas. M. Day, Aiken, S. C.; T. W. Hentz, Columbus, Geo.; H. A. McDaniel, D.D.S., Huntsville, Ala.;

F. Y. Clark, M.D.,D.D.S., Savannah, Geo.; C. A. Jordan, Huntsville, Ala.; H. D. Boyd, Troy, Ala.; J. G. McAuley, Selma, Ala.; J. D. Thomas, Atlanta, Geo.; W. J. Burr, M.D., Madison, Geo.; T. J. Crowe, Macon, Geo.; B. F. Arrington, D.D.S.,M.D., Wilmington, N. C.; Albert Hape, D.D.S., Atlanta,Geo.; F. J. S. Gorgas, D.D.S.,M.D., Baltimore, Md.; E. B. Marshall, Atlanta, Geo.; C. D'Alvigny,

Atlanta, Geo.; E. M. Allen, Marietta, Geo.; Wm. Reynolds, Columbia, S. C.; L. Augspath, Helena, Ark.; B. B. Alfred, La Grange, Geo.; Jno. Fouchè, Knoxville, Tenn.; W. H. Cooke, Cleaveland, Tenn.; Jacob Fogle, Columbus, Geo.; W. T. Cole, Newnan, Geo.; Jno. G. Angell, D.D.S., New Orleans, La.; Geo. J. Friedrich, D.D,S.; New Orleans, La.; L. D. Carpenter, Atlanta, Geo.; J. T. Campbell, Atlanta, Geo.; J. R. Cleveland, Griffin, Geo.; J. W. Wiley, Newnan, Geo.

The delegates having signed the constitution the meeting was called to order and the election of officers and Committees being next in order, resulted as follows, after much ballotting:

*President*—Dr. W. T. Arrington, of Tenn.; 1*st Vice President*—Dr. W. Reynolds, of S. Carolina; 2*d Vice President*—Dr. Augspath, of Arkansas; 3*d Vice President*—Dr. J. G. McAuley, of Alabama; *Corresponding Secretary*—Dr. F. J. S. Gorgas, of Maryland; *Recording Secretary*—Dr. John G. Angell, of Louisiana; *Treasurer*—Dr. W. G. Redman, of Kentucky; *Executive Committee*—Dr. W. H. Morgan, of Tennessee, *Chairman;* Dr. J. S. Knapp, Louisiana, Dr. W. S. Chandler, Louisiana; Dr. J. R. Walker, Louisana; Dr. A. Hape, Georgia.

The officers being duly installed, the president stated that the meeting was ready for business.

On motion of Dr. Redman the Association went into an election of the place most convenient and suitable for the next annual meeting.

Memphis, Nashville, Savannah, Augusta, Baltimore, Columbia, Montgomery and New Orleans were recommended and strong claims urged for each, but after due discussion, all were withdrawn in favor of New Orleans, La.

Committee on Constitution and By-Laws were continued until next annual meeting.

The Corresponding Secretary read communications from Drs. J. A. Thurber and A. F. McLain, of New Orleans, La.; W. H. Shadoan, Louisville, Ky.; W, L. Burton, Sec'y Richmond D. A., and W. W. H. Thackston, Farmville, Va., all expressing their regrets at not being able to attend the convention, and the assurance that we had their warmest sympathies and best wishes for success. The letter of Dr. W. W. H. Thackston was ordered to be spread upon the minutes of the meeting.

MORNING SESSION, THURSDAY 29TH.

Meeting was called to order by Dr. W. T. Arrington, the President in the chair.

No other business occupying the attention of the meeting the President appointed the following Committees:

*On Membership*—Drs. J. S. Knapp, La.; T. J. Jones, Ga.; G. J. Friedricks, La.

*On Publication.*—Drs. W. S. Chandler, La.; J. R. Walker, La.; J. G. Angell, La.

*Dental Education.*—Drs. F. J. S. Gorgas, Md.; J. P. H. Brown, Ga.; W. M. Reynolds, S. C.

*Physiology and Surgery.*—Drs. F. Y. Clark, Ga,; S. Rambo, Ala.; John Fouchè, Tenn.

*Dental Chemistry.*—Drs. J. G. McAuley, Ala.; W. H. Burr, Ga.; E. M. Allen, Ga.

*Histology and Microscopy.*—Drs. W. T. Arrington, Tenn. T. J. Jones, Ga.; John G. Angell, La.

*Dental Therapeutics.*—Drs. F. Y. Clark, Ga.; G. J. Friedericks, La.; H. Marshall, Ga.

*Operative Dentistry.*—Drs. W. H. Morgan, Tenn.: J. Fouchë, Tenn.; H. A. Lowrance, Ga.

*Mechanical Dentistry.*—Drs. W. G. Redman, Ky.; E. W. L'Engle, Ga.; S. G. Holland, Ga.

*Dental Literature.*—Drs. J. P. H. Brown, Ga.; H. A. McDaniel, Ala.; T. J. Jones, Ga.

*Voluntary Essays.*—Drs. J. R. Walker, La.; J. M. Day, S. C.; W. S. Chandler, La.

The President having appointed the various committees and the election of members being in order, the following gentlemen were elected honorary members of the Association:

Drs. S. P. Cutler, Holly Springs, Miss.; R. Arthur, Baltimore Maryland ; W. W. H. Thackston, Farmville, Va.; T. B. Hamlin, Tenn.; Profs. Samuel Gross, Philadelphia, Pa.; Warren Stone, New Orleans, La.; Paul F. Eve. Mo.; S. H. Stout, Geo.

Balloting for membership being closed, Dr. Morgan read a letter from Dr. J. F. Cannine relating to his experience in Rose Pearl for Plate work. He wished other dentists to be benefitted by his experience, as Rose Pearl did not serve the purpose represented by the author. The communication was received and referred to Committee on Mechanical Dentistry for further action.

The Secretary was then instructed by the chair to furnish each member of the various committees a list of the names of the committee to which they belong with the proper heading.

Dr. J. P. H. Brown of Augusta, Geo. read an able Essay on the present status of the Dental Profession, which was received and referred to Committee on Voluntary Essays.

Dr. W. H. Morgan, of Nashville, could not agree with Dr. Brown in two or three of his statements. He did not believe in that inate tact he referred to, and ignored the idea of a man being born a Dentist. The development of a man's mind depended upon his education. That some men may have a greater ability for one thing than another he did not doubt, but that was the result of the brain. He did not consider rubber a curse to the profession, it was rather a blessing, as it was the means of giving them an opportunity of developing the resources of the mind.

Dr. Day, said he was afraid Dr. Morgan had not understood his remarks of the previous day. He "meant" that some men were better suited to one position than another, that some men could not make good dentists if they tried all their life, such men had better turn their attention to something better suited to them.

Dr. B. F. Arrington, was much pleased with Dr. Brown's paper, was always glad to hear scientific articles, but he regretted the latter part of his article, and hoped it would be erased, if it should be published. He did not think there were too many Dental Colleges, if they were properly located. He had conversed with Professors of all the Colleges except St. Louis, and he believed it was our duty to bear with these institutions and not discourage students from attending them, but encourage them to take a degree in some college; the time was coming when it would be necessary to have it.

Dr. Brown, could not see any objections to anything in his essay, thought we would be better off, if there were fewer schools, the community could better support them. The professors would be better paid, and they would devote more time to lecturing than when not properly remunerated. The schools could have a more extensive museum and chemical aparatus. A man cannot be a dentist unless he has some mechanical talent, and some men may possess talent in a higher degree than others.

Dr. Morgan, said that the Association did not endorse all papers received and was not responsible for what they contained. Thought the number of schools were an advantage rather than a disadvantage. In 1847 there were only two schools with a very few scholars in the United States. Now there were eight schools and over five hundred students. He believed every man with a sound mind had some mechanical turn, and that it did not require a man of superior genius to become a good operator.

Dr. Brown, replied that in 1847 only two schools being in existence was owing to the low state of the profession.

On motion of Dr. Morgan the discussion was closed.

Dr. Jas. S. Knapp, read an Essay from Dr. A. F. McLain of New Orleans, La., on Prophylaxis or Prevention to Dental Decay.

On motion of Dr. Angell it was received and the subject discussed.

Dr. Reynolds of South Carolina, said he had never listened to a paper with more satisfaction. It was filled with truth, and nothing could be added but the easiness these principles could be inculcated to the parents.

If these ingredients that go to making teeth are not eaten, the childs teeth will be made from the mothers. Give the child corn bread, unbolted wheat bread or barly. If we submit to this diet during gestation of the mother and bring up children upon it they will have good teeth. The Scotch have good teeth because they have nourishing food.

The discussion ceased for a moment as the committee on Photographs reported that it would cost $6 for fifty carte size for each member.

On motion of Dr. Knapp the report was referred back to the Committee with instructions to act upon the group.

On motion of Dr. B. F. Arrington it was resolved that the ladies who had favored us with their presence and Mr. Hape be included in the group.

The discussion on Dr. McLain's paper was resumed. Dr. J. R. Walker of New Orleans thought it was an able paper, considered it our duty not to repair damages but to go to the origin of the difficulty and see if our patients had proper food, knows of several instances where improvements have resulted by mothers eating nutritious food. Dr. Morgan said children must be cautioned against biting hard substances. He thought they must be cautioned against not biting. They must masticate their food and give exercise to the teeth.

Dr. F. Y. Clark, said he was much pleased with the essay, but that we must know the cause of the disease before we can remove it. He believed the real cause of diseased teeth was frequently from improper development of the child, from contracted jaws in children, extending sometimes through generations. If parents had no teeth the children would probably be the same. Indians had good teeth; their infants were perfect, jaws well developed about 1 or 1½ inches more than our race. Field hands among the negroes were the same. But in the kitchen bad teeth are caused by the acid in the saliva and hot food. Animals fed upon the refuse of distilleries and turned out to pasture would die, because they had not the teeth to masticate. Emigrants are well developed and have fine teeth. Fashionable circles have artificial teeth and deformed jaws, quite contrary to the more humble class. Felt it ought to be made plain to the people, that nutritious food, proper cleanliness and use are essential to good teeth. Thought that defect in many teeth was caused by Physicians lancing the gums of children teeth.

Dr. Knapp thought when children teeth were ready to erupt, the enamel was too hard to be defaced or indented by the lance and that the defect referred to by Dr. C. was caused by sickness of the child during the formation of the teeth.

Dr Reynolds coincided in Dr. K's views.

Dr Clark said the crack or mark left by the lance was different from that of sickness and could scarcely be observed at first.

Dr. McDaniel considered the subject of great importance and felt we should give it special attention as it had been to entrusted to the medical adviser.

Dr. B. F. Arrington wished to express his appreciation of the paper, but thinks we were going behind our profession. Thinks the medical fraternity ought to attend to the constitution of the patient, our duty is to prepare ourselves for treating these diseases when they appear. Believed that it is our duty to watch over children's teeth entrusted to our care.

Dr. Day felt much interest in the paper and was confident it contained much truth as he had seen the benefit of its advice to some of his patients.

On motion of Dr. Morgan the discussion was closed.

Dr. Gorgas read a deep and scientific essay on "Microscopy of the Teeth," by Dr. S. P. Cutler of Holly Springs, Miss. On motion of Dr. Knapp the thanks of the Association were tendered to Dr. Gorgas for the able manner he read the article.

Committee on Photographs reported that pictures of the group would be taken by the artist 11 x 14 at 9 o'clock Friday morning at $2.50 each.

Dr. Clark displayed a peculiar piece of mechanical plate work, said to have been worn by General Oglethrope. The meeting udjourned to meet at 8 P. M.

NIGHT SESSION, THURSDAY 29TH.

Meeting was called to order as per adjournment at 8 P.M. by Dr. W. T. Arrington, the President, in the chair.

Dr. Knapp moved to adjourn this session of the Association at the hour of 2 P. M. Friday, July 30th, 1869. Carried.

On motion of Dr. B. F. Arrington, Dr. Knapp was appointed a committee of one to draft complimentary resolutions to Mr. Hape.

On motion of Dr. McAuley the subject of alveolar ab-scess, fang filling and necrosed teeth was discussed.

Dr. Clarke being called upon for his experience, for brevity sake confined himself to three cases which he thought would embrace all the others to a certain extent.

The first, is where the nerve has suppurated but no abscess. He had found more trouble with this class than with any other. It was easy enough to remove the nerve and fill the root, but the mephitic gas always gave trouble on being confined. Never could get satisfactory answers to his investigations. He supposed trouble was caused by using unsuitable instruments and clogging up the foramen. He usually treated such cases by syringing out the cavity thoroughly the first sitting, wiping it out with creosote the second, and filling the third, taking care not to stop up the foramen with loose decay.

Second class, is where there is a fistulous opening, they are generally simple and no trouble to treat. He syringes out the first sitting and removes as much decay as possible, taking care not to fill up the foramen, then with the best instrument wrapped with cotton, pump creosote into the cavity until he sees it come out of the fistulous opening. Continue this treatment until the tooth is in a healthy condition, then fills the root to the foramen with gold taking care not to force it through.

Third class. The nerve is not entirely destroyed is very sensative, bleeds freely. He removes the nerve and after twenty-four or forty-eight hours applies creosote, and at third sitting fills the cavity.

Dr. B. F. Arrington; thinks bees-wax is the best material in the root of a tooth, it flows where you cannot put anything else, it is indestructable and fills the entire cavity. Next to wax is Os-Artificial, considers this better than cotton.

Dr. Augspath of Arkansas, said he had used Os-Artificial in his practice for eight years past for root filling and had been very successful.

Dr. Morgan referred to diseased teeth in the same order as Dr. Clarke. He agrees with Dr. C., that with the first class, there was always after trouble. He removes all decay and treats with creosote. Thinks trouble is caused by clogging up the foramen. When seriously apprehending an abscess following his first operation he applies the compound tincture of Iodide of Potassium on the gum over the root inside and out, treats sometimes several weeks. Sometimes fills the root with wood but prefers gold.

Second class. There is but little vitality, portion of the pulp has sloughed off, and little spongy growth which bleeds profusely. Has less apprehension with these cases than with No. 1. He devitalizes the pulp and fills the root. In Alveolar abscess prefers carbolic acid to creosote. He has met with few cases he could not control with any remedy. When there was yellowish discharge he had never succeeded in controlling.

Considers chances of success in strumous habits less liable than in bilious or sanguine temperaments Treatment is also modified by age, more difficult in young than in the old. The six year old molars up to fifteen years when the other teeth are good never devitalizes, but removes the tooth as the gap will close up.

Dr. Rambo, finds same difficulty, as Drs. M. and C. They are apparently easy, but when operated upon we have violent inflammation insuing. Sometimes thinks he forces air through the foramen. Fills with gold avoiding forcing it through the foramen. Treats fistulous opening and then fills same as others. Where nerve is not entirely devitalized, as shown by cold and warm drinks producing pain, he destroys and fills his usual way.

Dr. Brown of Augusta. If in the 1st case there is a discharge through the decay, the trouble to treat depends upon the constitution.

He arrests the discharge if possible and removes the dead pulp as much as possible, and wipes out the root with creosote, then fills the root for a few days with sadler's silk saturated with creosote, then removes and if it is impure he applies a fresh piece. Fills with cylinders. Thinks trouble arises from not filling the root well. If the gold protrudes periostitis will ensue, recommends in such cases Aconite and Iodine, equal parts, internally and externally.

Dr. Alfred of Georgia, said he had used the preparation of Aconite and Iodine with great success.

Dr. Jordan of Alabama, used floss silk saturated with creosote to fill roots, then fills crowns with gold.

Dr. Walker of Louisiana, prepared roots by thoroughly treating canals with carbolic acid until all soreness is gone, which may take days or weeks, he then fills with cylinders wrapped on a broach rather harder than those used in crown fillings. In treating root he introduces the cotton on a small piece of wood, and if the canal is sufficiently large he obtains its form with a piece cotton. Fills the root with plaster of paris as follows: With a syringe composed of a quill having a flexible ball of rubber on one end and a fine nozzle on the other, after introducing creosote as before stated, force dry plaster of paris up the canal, the transparency of the quill enabling one to determine how much plaster is introduced. The entire canal is filled in this way with a hard gray cement.

Dr. Friedrichs considers that such cases are only successful when nature steps in and remedies the evil. He never fills the root until nature has performed her part, and believes that filling the apex of the root is sufficient without filling the balance of the canal, and filling over an exposed pulp does not necessarily lead to its death. Cleans the canal with a pellet of cotton, wrapped on a broach, saturated with water and dipped in pumice. Uses gold to fill the canal. Objects to Os-artificial as it sets too rapidly.

Dr. H. Marshall said he always fills roots with gold. Never fills teeth directly after devitalizing the pulp, prefers treating eight or ten days, and thinks we only assist nature in such cases. Treats alveolar abscess with tannic acid, camphor and tincture of iodine, and in a week or ten days fills root with sponge gold dipping the first used in creosote.

Dr. L'Engle considers filling with wood impracticable in every case.

Dr. Holland described a case of three and a half years standing, he removed the filling and on passing up a broach found pus in the canal, he treated with diluted tincture of iodine for six weeks, then filled with gold, introducing it with the finest watchmaker's broach, winding a strip of gold around it, first dipping the end of the gold in creosote. Uses annealed and partial annealed broaches.

Dr. Redman had nothing new to offer, was not as successful as some others, wished to know how to get out of trouble when he is unsuccessful. In alveolar abscess he pierces to point of root and treats externally through alveolus, sometimes successfully and sometimes fails.

Dr. Ford. Fills roots with gold about two-thirds and then a layer of Hill's stopping and finishes with gold. Thinks interposing a non-conductor an advantage.

Dr. Carpenter fills with cylinders, believes cotton to be injurious.

Dr. Gorgas considers the use of a non-conducting substance an advantage. Uses Hill's stopping with os-artificial. The first at the apex and os-artificial on the Hill's stopping until he reaches the crown cavity, thus preventing the chloride of zinc from acting at apex of the root. Prefers using gold in in the form of small pieces torn from the sheet for filling pulp canals. He first determines the length of root and diameter of canal and introduces the gold as far as it can be forced with safety. If the canal is large prefers gold foil in the form of strips, folding one over the other as he fills the root. Treating alveolar abscess feels we have much yet to learn. Has used carbolic acid, iodine and glycerine successfully. Had a case about one year since, superior lateral incisor, fistulous opening extending from apex of root to near the second bicuspid. Injection passed through opening, it was of some eighteen months standing, drilled an opening over the root on the labial surface, and the fistulous opening closing, the discharge continued through the labial opening; treated successfully with carbolic acid, and iodine and glycerine.

Dr. McAuley, treats with iodine, creosote and aconite, does not know whether nature or the medicine effects a cure. If these applications are made succcesfully the next morning, the patient appears and nature meets your efforts with a smile, and if you do not succeed, a frown greets you and that frown is nature's face. He always fills with gold.

Dr. B. F. Arrington never fills a root.

Dr. Augspath advocates using os-artificial in root filling, introduces it with a bristle, and to prevent it setting too rapidly in summer keeps it on ice.

Dr. Morgan considers it important to fill roots to the apex. Objects to using fine broaches as they may pass through the foramen.

Dr. Gorgas said he used an instrument for the removal of the sac through a fistulous opening, made from a piece of old watch spring.

Dr. J. G. Angell said the chances of success depended a great deal upon the temperament of the patient. Some patients might have their tooth filled without removing the dead nerve and not suffer the least inconvenience, while another having received every attention and care that ingenuity and skill could command, yet trouble would ensue in less than twenty-fours after the operation was completed, he had been using for several years past in treatment of alveolar abscess, the tincture of hydrastis canadensis and found it much more effective in relieving and curing than any other remedy yet known.

The discussion being ended the meeting adjourned until 9 o'clock the following morning.

MORNING SESSION, FRIDAY 30TH.

The meeting was called to order at 9 A.M. by Dr. W. T. Arrington, the President, in the chair.

On motion of Dr. Jordan the reading of the minutes of the previous meetings was dispensed with. On motion of Dr. Carpenter the subject of exposed pulps was introduced for discussion.

Dr. Clark having the floor, stated that his treatment of exposed pulps was very simple, if he wished to destroy the pulp he made an arsenious application on a small plegget of cotton, then to avoid pressure placed a second and larger piece over that, leaving it in form 12 to 24 hours, then if he could remove the nerve and no unfavorable symptoms were present, he filled at once, but if there were any unkind symptoms, such as profuse bleeding or irritability, he delayed filling until by proper treatment all such symptoms disappeared. In simple exposure of the pulp there is no inflammation. He never attempts to remove until he has failed to save by inducing ossific deposit. This is a subject to which he has lately given much attention, and one which he thinks worthy of much more. He recited one or two cases to prove that ossific deposit is no longer a matter of doubt, but a fact.

Dr. Friedrich always treated to preserve pulp if possible and considers it our first duty. If it is healthy it requires no treatment. If there is hæmorrhage he arrests it by cauterising pulp with nitrate of silver, and thus forms a thin cuticle or skin to protect it. Does not consider the tooth is decomposed in the least by a slight application of nitrate of silver. Caps the nerve with some non-conducting substance and fills the tooth immediately.

Dr. B. F. Arrington caps nerve with asbestos wrapped in gold or tin foil, and has been very successful for the last six or eight years.

Dr. Walker mentioned a case he had several years ago. Superior central incisor, large cavity and large exposure of pulp, the patient was anxious to save pulp. He caped it with vulcanite base, strong enough to sustain pressure of a gold filling. He has used os-artitificial subsequently with better success than the vulcanite base. Considers preserving the pulp of vital importance, would attempt it if there was one chance out of five of saving it, and the patient was sufficiently intelligent to appreciate it.

Dr. Knapp considers nothing more desirable than to preserve the vitality of the pulp. In the last two or three years some changes have taken place in the treatment of pulps by some of our best operators, but does not think sufficient time had elapsed to judge well. He has had but limited experience in treating pulps, has followed Dr. Atkinson's method by flooding the cavity with creosote and capping with os-artificial and been quite successful. In some instances great pain is produced by os-artificial, requiring removal in a few minutes and devitalizing with creosote, and returning the application in the cavity with cotton saturated with gum sandarac, Thinks pain is produced more by the pressure than by the application itself.

Dr. Rambo said he had met with so little success in preserving the vitality of pulps that he does not undertake it except in few cases. To devitalize, uses arsenic, ac-morphia, creosote and glycerine, protects application by lead cap, it prevents pressure. Had used collodion and sandarac.

Dr. Morgan had been in the habit for the last twenty years of devitalizing and removing the pulp. He had tried all plans of capping. He had better success with tin than with gold, but preferred os-artificial to them all. Had little confidence in saving pulp, where there is exposure and bleeding. To devitalize uses arsenic and creosote, sometimes uses arsenic and water, and thinks it is the proper mode of using it.

Has been successful in capping with cs-artificial. If there is an excess of fluid it will produce pain, the cause of the pain is the chloride of zinc. Tooth is sometimes red from broken up red globules and the coloring matter of the blood being injected into it.

Dr. Gorgas used creosote and ac-morphia to destroy pulp. Creosote is the vehicle; has used water with success. Does not believe in using caps over nerves. Has been successful with os-artificial, applies it on old or soft linen and fills the crown cavity with the os-artificial and allows it to wear away and then fills with gold.

On motion of Dr. B. F. Arrington the regular order of business was suspended to hear a paper read by Dr. Crowe as follows:

"A gentleman in fine health about thirty years of age applied to me about a year ago for treatment. Upon examination I found the right superior lateral incisor moved forward from its natural position from the habit of holding the pipe under it in smoking. It was easily moved in its socket, and there appeared to be a wasting of the gum around it, the root being somewhat exposed. There was also a yellow deposit in a slight degree, of a soft matter somewhat offensive in odor. The patient was exceedingly cleanly in habit, and his teeth remarkably free from decay. My treatment was first to move the tooth to its original position with a rubber band. The patient then gave up smoking and used an antacid mouth wash. Three weeks after there was no improvement whatever.

The treatment was then changed to the use of acids and careful regulation of the diet. Still no improvement. Some months after, during my absence, the tooth was extracted by a brother dentist, and on my return was brought to me for inspection. It was perfectly sound, the fang, however, I found to be covered on one side by a dark brown incrustation having every appearance of salivary calculus. It appeared to be uniform in thickness throughout its whole length, and extended to within about three lines of the apex of the fang. Would it have been advisable after the extraction of this tooth and the discovery of the deposit thereon to have replaced the tooth in its original position?

Dr. Morgan said it was a bad case of salivary calculus. Sometimes whole families were affected. An old gentleman and all his family have salivary calculus on their teeth, treated them successfully by removing the gum up to apex of the root and removing the calculus, there was not so much sponginess as in some other forms of disease, there was a blueness of the gum, he always in such cases removed the pocket up to the beginning of the periosteum, and treated with carbolic acid, it being both an astringent and stimulant. Had a case fourteen years ago where he could run the instrument up to the apex of the root, he removed the pocket and exposed the root up to the apex. Knew that Dr. Robinson, the first male borne in Nashville, transplanted successfully some sheeps teeth in his family.

Dr. Knapp said tartar deposited under the gum irritated it and produced absorption, he always removed it with instruments and sometimes recommended astringent mouth washes to harden the gums.

Dr. Clarke said he did not think tartar existed on the root in the case referred to by Dr. Crowe. There could be no absorption where there was no tartar; he could not say what the deposit was.

Dr. Walker said he had seen cases of absorption without tartar, generally confined to teeth that hold the pipe or cigar when smoking. Thinks nicotine may be the cause.

Dr. Morgan said all crystaline formations were tartar.

Dr. W. T. Arrington said he had seen cases where it was not calculi but denuding of the periosteum from nicotine.

On motion of Dr. B. F. Arrington, the Code of Ethics was read for the benefit of the various members of the Association.

A resolution of thanks was tendered to the city, the press, the various hotels and Railroad Road companies, for favors and civilities generously extended to the Association.

The following resolutions were unanimously passed.

*Resolved.* That this Association will not at this or at any future meeting as a body, take into consideration and report upon the merits or demerits of dental material, instruments or furniture of any description.

*Resolved.* That the Southern Dental Association hereby most respectfully tenders a vote of thanks to Mr. Samuel Hape of the Dental Depot, of Atlanta, Ga., for his successful efforts in making excellent arragement for their first meeting and for the many civilities extended to them during the session.

Dr. John G. Angell of New Orleans read a thesis upon the subject of Anæsthesia which was accepted and referred to the proper committee.

The following members were delegated to the American Dental Association.

Drs. Morgan, Gorgas, Walker, Clarke and Crowe.

The printing of the Code of Ethics was referred to the Publication Committee.

The subject of Green Stains was then introduced for discussion, and Dr. Rambo being called upon said that he observed these stains from infancy to puberty and thinks it is the effect of softening of the enamel by acids, caused by want of proper cleanliness, producing disintegration of the enamel. If there is no decay he removes it with pumice and soft wood, and polished with soap. It will return again unless the child takes great care of the teeth. He does not like to remove it with instruments.

Dr. Clark had observed these stains with sensitiveness of the teeth, and gum receeding from the enamel. He removes the stains in a similar way to Dr. Rambo, and treats the sensitiveness with one or two applicatious of the nitrate of silver. If it discolors the tooth it can be restored by applying salts or chlorine.

Dr. B. F. Arrington said he had been using nitrate of silver twelve or fifteen years with success. He envelopes the caustic with wax and trims it according to the surface he wishes to apply it. If to be applied to the neck of the tooth, he wipes the gums dry and covers with a film of collodion. The discussion was then closed. Mr. Sam'l Hape was instructed to forward to each member of the Association one or more copies of the paper containing an official account of the proceedings of the meeting of the Association, and forward the bill to the Treasurer Dr. Redman.

The Executive Committee was instructed, by vote of the Association, to prepare a cane, with gold head and proper inscription, to be presented to Mr. Samuel Hape at the next annual meeting to be held in New Orleans.

Dr. McAuley being called upon for the closing remarks, said he was glad he had come to the meeting. He had left his office to take care of itself, but felt he was amply repaid for the sacrifice, and hoped we would all meet face to face in New Orleans next April. All the other members coincided in the remarks of Dr. McAuley, and with good spirit and congenial feelings the Association adjourned to meet the second Wednesday in April next at New Orleans, La.

---

